# Strengthening clinical trials in African primary care

**DOI:** 10.4102/phcfm.v16i1.4677

**Published:** 2024-07-23

**Authors:** Robert Mash

**Affiliations:** 1 Division of Family Medicine and Primary Care, Faculty of Medicine and Health Sciences, Stellenbosch University, Cape Town, South Africa

In 2022, the 75th World Health Assembly made a resolution to strengthen ‘clinical trials to provide high-quality evidence on health interventions and to improve research quality and coordination’.^1^ In June 2024, I was invited to a stakeholder consultation at the World Health Organization (WHO) in Geneva to try and develop an action plan in response to this global commitment.

One of the key challenges is the relative lack of clinical trials in low- and middle-income countries (LMICs) despite the high burden of disease.^2^ We know that the total number of clinical trials in Africa is very low as a proportion of trials globally and within this primary care research is even more limited. A recent review of research published in the two leading family practice journals in Africa found that only 8% of publications came from experimental study designs, and these were mostly quasi-experimental and before-and-after type studies.^3^ There is a dearth of clinical trials within African primary care and family medicine.

Clinical trials address important questions of safety and effectiveness and allow us to evaluate whether interventions have beneficial health outcomes. Typically, such trials focus on drugs and medical products, but can also include behavioural interventions and service delivery. One of the problems is that many clinical trials are uninformative or unhelpful. For example, less than 10% of coronavirus disease 2019 (COVID-19)-related clinical trials produced results that were useful for decision making.^4^ Similarly, of all new medicines approved in France between 2013 and 2022, based mostly on industry-related clinical trials, only 10% demonstrated ‘a real advance’ or offered ‘an advantage’ over existing medication.^5^ There is therefore a lot of waste in clinical trials that are underpowered, poorly designed or asking unhelpful questions. Many trials also fail to include or target pregnant women, children, and older adults, leaving clinicians without an adequate evidence base.

In primary care, we often rely on extrapolated evidence from hospital or specialist environments that may be inappropriate. Primary care, however, needs its own evidence and can address its own unique questions. Patients in primary care may be seen earlier in the course of the disease, may present with undifferentiated problems and diagnostic dilemmas, may be seen at a time when prevention is possible, and may include people and conditions rarely seen in hospital settings. Primary care requires a wholistic approach, and clinicians frequently have questions related to person-centredness, empowerment, lifestyle modification and support of self-management. Primary care research can include basic, clinical, health system, health services, and educational studies.^6^ While basic research might develop the tools to use in clinical trials, interventions might be considered for evaluation in all the other areas. There is also a need to focus on interventions to improve the implementation of primary healthcare that includes population health management, community empowerment and multisectoral action.^7^

Why are so few clinical trials conducted in African family medicine and primary care? Currently, much of the research output is generated by novice researchers doing master’s level research.^7^ There is a small, but growing number of early career researchers with doctoral degrees or equivalent track records. There are very few established researchers with the expertise to compete for international clinical trial funding and to create and lead multicentre or multicountry teams. At the same time, there is inadequate national level funding for clinical trials and a neglect of primary care research in most countries. Regulatory bodies and ethics committees often impede the preparation of clinical trials through multiple, lengthy, complex and sometimes contradictory processes.

The WHO Global Clinical Trials Forum is developing recommendations for action around 10 key issues as shown in Table 1. One of the ideas in the meeting was that of ‘disease-agnostic and always-on’ clinical trial units or networks. Primary care is an appropriate context for a network to be disease-agnostic and to address a wide variety of diseases from different parts of the life course. Currently most clinical trial units focus on specific diseases or groups in an approach that mirrors the specialist hospital environment and a verticalised fragmented health system. The other idea is that a clinical trials network that is ‘always-on’ can build up the expertise and capacity to perform trials. In once-off funding approaches, this expertise is often hard won during the trial and lost once the funding ends. Within such a network, a variety of trials could be at different stages of preparation, data collection, analysis and reporting. Such a network can be more responsive and adaptive to urgent issues such as seen during COVID-19.

**TABLE 1:** Proposed actions to strengthen clinical trials.

No	Action
1	Strengthen local leadership and national support for sustained infrastructure and funding
2	Enhance engagement with patients, communities and the public in trial life cycle
3	Address barriers to clinical trials in under-represented populations
4	Ensure trials are well designed including adoption of innovative designs and digital technologies
5	Accelerate access to fit-for-purpose training packages for clinical trials
6	Improve coordination and streamlining regulatory and ethics review
7	Engage clinical practitioners to integrate clinical trials into health systems and practices
8	Step up the use of trial registries for research outcome reporting
9	Expand international health research and clinical trial collaboration
10	Identify exemplar trials to monitor how reforms can accelerate generation of quality evidence

Primary care has developed many practice-based research networks that could fulfil such a function if adequately resourced and capacitated.^8^ Such networks have, as a premise, the integration of research into clinical practice and health services. In Africa, such networks are unusual, but do exist in South Africa,^9^ and in the Primary Care and Family Medicine (PRIMAFAMED) network.^10^ The PRIMAFAMED network has a history of collaborative multicountry research projects and includes 25 African countries,^11^ although it has never performed a clinical trial. Looking at the recommended actions, it is also obvious that primary care clinical trials can enhance engagement with patients and communities as well as under-represented populations. Family medicine and primary care in Africa would benefit from South–South–North collaboration to develop the expertise and access funding. Our challenge to the WHO Global Clinical Trials Forum is to ensure that at least one of their exemplars (see action 10 in Table 1) is in primary care.
